# Mental Fatigue-Associated Decrease in Table Tennis Performance: Is There an Electrophysiological Signature?

**DOI:** 10.3390/ijerph182412906

**Published:** 2021-12-07

**Authors:** Jelle Habay, Matthias Proost, Jonas De Wachter, Jesús Díaz-García, Kevin De Pauw, Romain Meeusen, Jeroen Van Cutsem, Bart Roelands

**Affiliations:** 1 Human Physiology and Sports Physiotherapy Research Group, Faculty of Physical Education and Physiotherapy, Vrije Universiteit Brussel, Pleinlaan 2, 1050 Brussels, Belgium; jelle.habay@vub.be (J.H.); matthias.proost@vub.be (M.P.); jonas.de.wachter@vub.be (J.D.W.); kevin.de.pauw@vub.be (K.D.P.); romain.meeusen@vub.be (R.M.); Jeroen.Vancutsem@mil.be (J.V.C.); 2 BruBotics, Vrije Universiteit Brussel, 1050 Brussels, Belgium; 3 Faculty of Sport Sciences, University of Extremadura, 10003 Caceres, Spain; jdiaz@unex.es; 4 Vital Signs and Performance Monitoring Research Unit, LIFE Department, Royal Military Academy, Avenue de la Renaissancelaan 30, 1000 Brussels, Belgium

**Keywords:** mental fatigue, table tennis, electroencephalography, event related potentials, visuomotor performance

## Abstract

Mental fatigue (MF) is a psychobiological state negatively impacting both cognitive and physical performance. Although recent research implies that some table tennis (TT) performance outcomes are impaired by MF, open skill sports such as TT require a more detailed overview of MF-related performance decrements. Moreover, research into MF and sport-specific psychomotor performance lacks the inclusion of brain-related measurements to identify MF mechanisms. Eleven experienced TT players participated in this randomized counterbalanced crossover trial. Participants were either required to perform an individualized Stroop task (MF condition) or watch a documentary (control condition). The primary outcomes were reaction time on a sport-specific visuomotor task and EEG activity throughout the trial. The subjective feeling of MF was significantly different between both conditions and confirmed that the MF condition induced the mentally fatigue state of participants (*p* < 0.001), though no behavioral indicators (i.e., decrease in performance on Stroop and flanker task) of MF. MF worsened reaction time on the visuomotor task, while other secondary measurements remained largely ambiguous. Spectral power (i.e., decreases in upper α band and θ band) was influenced by MF, while ERPs measured during the visuomotor task remained unaltered. The present study confirms that MF negatively impacts table tennis performance, specifically inhibitory stimuli during the visuomotor task. These findings also further augment our understanding of the effects of MF on human performance.

## 1. Introduction

In elite sport performance, athletes use both advanced cognitive and motor skills to win games, advance in world rankings and earn trophies. Moreover, in elite athletes, it is often differences in cognitive skills such as decision making [1], visuomotor reaction time [2] and attention [3] that decide winning or losing [4,5]. This is especially true in fast sports such as table tennis (TT), which consist of very specific physiological and physical demands [6,7,8] and a high contribution of cognitive resources [9]. These cognitive demands can be linked to the immense time pressure of the game [9] and the need to stay focused in an open sport environment [10,11]. Research has also shown that TT athletes display faster visuomotor reaction times [9,12], a higher level of executive functions [13], better unconscious response inhibition [11] and greater dual task performance [14] compared to non-athletes, novices or even medium-level TT players. However, this important contribution of cognitive skills is not exclusively beneficial, as this may lead to a higher mental load on the individual, which can result in a higher risk of suffering decrements in performance due to mental fatigue (MF) [15]. Therefore, ideal cognitive capacity for TT athletes is needed to optimally perform, and as such it is of the utmost importance to identify and counteract MF as quickly as possible [16].

MF can be defined as a psychobiological state that emerges during prolonged demanding cognitive activity and results in an acute feeling of tiredness and/or decreased cognitive skills [17,18]. Recently, multiple researchers in sport science have directed their attention towards MF and its effect on multiple types of performance [19]. One study has already identified reduced TT performance due to MF [16], observing a negative effect of MF on ball speed, accuracy of the ball, number of faults and total TT performance. This study gave important insights into the detrimental effects of MF on sport-specific psychomotor performance, and remains the only study, to the best of our knowledge, that examined the effect of MF on TT related skills [16,18]. However, even though TT performance is negatively impacted by MF, some important measures of sport-specific visuomotor performance, such as reaction time, were not assessed by Le Mansec et al. [16]. An approach to measure reaction time could be the visuomotor test utilised by Van Cutsem et al. [20], who examined the effect of MF on visuomotor performance in badminton players. The utilisation of this visuomotor test could provide valuable additional information regarding the effect of MF on TT performance, since TT and badminton players are subjected to similar cognitive load while playing [21]. Moreover, and more importantly, the addition of brain-related measurements when assessing the effect of MF on TT performance could help explain the mechanisms behind MF-related decrements in sport-specific psychomotor performance, with the eventual goal of designing specific interventions to counter MF in elite athletes.

It is well established that MF impairs cognitive skills [22,23] and physical performance [17,18,24] in healthy individuals. Based on these decrements, mechanisms have been proposed to better understand this type of fatigue and to develop possible countermeasures. Proposed mechanisms include, but are not limited to, an increase in rate of perceived exertion (RPE) due to adenosine accumulation in the anterior cingulate cortex (ACC) [17,25] and the ego depletion theory (i.e., decrements in performance due to depletion of a global self-control resource [24]). However, these mechanisms of the negative effects of MF on performance remain mostly theoretical, as most sport science MF studies use circumstantial evidence (i.e., peripheral physiological and subjective outcomes) and fail to incorporate objective measures of the brain, such as electroencephalography (EEG) [18]. Particular EEG outcomes of interest include event-related potentials (ERPs) [26,27] and spectral power analysis (SPA) [28]. Regarding SPA, a recent review already showed the impact on theta and alfa activity due to MF, which are linked to decreased inhibition control and alertness [28]. Since alertness states have the potential to predict cognitive performance parameters, it could be argued that these changes in alertness are one of the drivers of the effect of MF on human performance [29]. The study of Van Cutsem et al. [30], detailing the influence of a caffeine-maltodextrine mouth rinse on MF effects, investigated the effect of ERPs on MF and found a decreased amplitude of the P2 component. This component is theorized to be a measure of, among other things, the allocation of attentional resources, a mechanism frequently associated with MF effects [30,31]. Other ERPs of interest in MF research include P3b [26], N2 [32,33] and N1 [32,33,34], with all of them connected to specific mechanisms of MF. However, the addition of EEG measures in MF research remains in its infancy, and studies investigating these measures during motor tasks are also rare [18]. Further investigating EEG outcomes under conditions of mental fatigue can give us valuable information on the possible mechanisms behind the effects of MF on sport-specific psychomotor performance [18].

Therefore, the primary aim of the present study was to investigate whether MF impairs sport-specific visuomotor performance in TT players. We hypothesised that MF would worsen reaction time, based on knowledge gathered by similar studies [16,20]. A secondary aim was to attribute possible differences in EEG activity between the MF and control condition to possible decreases in visuomotor performance. Our hypothesis was that MF would increase theta and alpha activity [28] while also decreasing the amplitude of ERPs of interest (i.e., N1, N2, P2 and P3b) during both the cognitive tasks and the TT performance task [26,27].

## 2. Materials and Methods

### 2.1. Participants

A total of 11 experienced table tennis players (4 ♀/ 7 ♂; age: 24 ± 2 yrs; height: 178 ± 10 cm; weight: 70 ± 10 kg; training volume: 7 ± 4 h/week) were included in this experiment. The table tennis level of participants ranged between A3 (elite) and C4 (trained). Participants were required to sleep a similar amount of time before each trial (at least 7 h), refrain from the consumption of caffeine and/or alcohol, and to avoid any vigorous physical activity the day before and the day of each visit. In addition, participants were asked to have a similar meal on the morning of each trial. The use of any kind of medicinal products during and between the trials was prohibited. If participants could not meet these standards, they were excluded from the study. Each subject gave written informed consent prior to the study. The present study protocol and its procedures were approved by the Research Council of the Vrije Universiteit Brussel.

### 2.2. Experimental Procedure

The study employs a randomized counterbalanced cross-over trial. Participants reported for the study on three separate occasions (familiarization, experimental condition and control condition) on the same time of day (in the morning) and with a minimum of three days between sessions. Measurements were conducted in thermoneutral conditions (20 °C, humidity 45%) and every trial took about two hours to complete. It was not specified to participants which was the control, and which was the experimental procedure. Participants were told that the goal of the study was to examine the effect of two different cognitive tasks on their TT specific reaction time and where, as such, blinded for our predefined hypotheses.

#### 2.2.1. Familiarization Trial

Participants were first invited to a familiarization trial where they had the chance to become acquainted with the different questionnaires (i.e., pre-test checklist, Mental Visual Analogue Scale (M-VAS), Matthews motivation scale, rating of perceived exertion (RPE), and National Aeronautics and Space Administration Task Load Index (Nasa-TLX)), the visuomotor task, and the experimental task (i.e., a Stroop task). The participants familiarized themselves with the experimental condition by performing a Stroop max test, which was designed to individualize the difficulty level of the Stroop test during the experimental trial. The circumference of the head was also measured for fitting the EEG cap during the experimental and control condition.

In the Stroop max task, four coloured words (“red”, “blue”, “green” and “yellow”) were presented one at a time on a computer screen. The participants were required to indicate the colour of the word, ignoring the meaning of the word itself. If the ink colour was red, the button to be pressed was the button linked to the real meaning of the word. The presented word and its ink colour were randomly selected by the computer (100% incongruent), with all incongruent word–colour combinations being equally common. The Stroop max test of the familiarization trial consisted of 108 stimuli in total, divided into three cycles of 36 stimuli with an average interstimulus time of 1500 ms. The level of difficulty on this test changed depending on the stimulus presentation time (i.e., the total amount of time a stimulus was on screen), ranging from 1200 ms to 500 ms with a difference between levels of 100 ms. Before the actual max test, subjects performed a warm-up to get acquainted with the cognitive task. Afterwards, participants commenced the Stroop max test at the 1200-ms level. If the participant was more or equal to 85% accurate (i.e., 85% of stimuli were correctly interpreted), they were allowed to proceed to the next level. If participants were less than 85% accurate, they had to repeat this level. Participants were allowed three attempts at a level and five attempts in total (in the case of multiple retries across levels). If a participant exceeded the amount of retries, the max test was concluded and the last level where the participant was able to meet the 85%-threshold was selected as their maximal cognitive performance level and was applied within the experimental trial.

#### 2.2.2. Experimental and Control Trial

**Figure 1:**
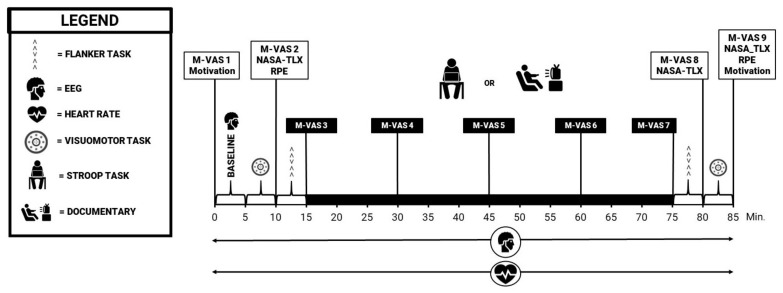
Schematic representation of the experimental and control trial.

The experimental and control trial proceeded in a similar manner, with the only difference being the intervention task in the middle of the trial. At the beginning of each trial the EEG device and heart rate monitor were attached, and a baseline EEG measurement was performed while the participant remained seated. Following the baseline EEG measurement, participants performed the visuomotor task and the flanker task. Participants were then instructed to perform either the intervention or the control procedure. Immediately afterwards the flanker task and visuomotor task were again performed. Throughout the measurements, participants were required to fill in a number of questionnaires (see Figure 1).

Flanker task: Participants were instructed to respond as quickly and accurately as possible to the direction of a target arrow while ignoring the direction of the other arrows on each side (e.g., < < > < <). Each array of arrows was focally presented in white text (font size 34) for 200 ms on a black background with a variable interstimulus interval of 1000, 1200, 1400, or 1600 ms. A total of 120 stimuli were given (15 cycles of 8 stimuli) with both right and left arrows appearing with equal probability. The total duration of the task was approximately 5 min. To assess performance on the flanker task accuracy and reaction time were collected. The aim of the flanker task was to make sure that participants were adequately familiarized and not already fatigued before the start of the intervention/control task [35].

Intervention procedure: The Stroop task was chosen to induce MF in the participants of the present trial. The instructions of the task were the same as the ones given when performing the Stroop max task. The differences between this task and the Stroop max task were the duration and the task specifications. The Stroop task of the intervention procedure had a duration of 60 min, which translates to 1500 stimuli that were divided into four blocks, with 360 stimuli every block and 90 stimuli for every colour. The time that the stimuli were presented was based on the achieved level on the Stroop max task. In between the four blocks, 15 stimuli were not taken into account for the performance analysis. This allowed the researchers to pose the question: “how mentally fatigued do you feel on a scale from 0 to a 100, where 0 means not fatigued at all and 100 means extremely mentally fatigued?”. The participants had no knowledge of the existence of the four blocks and received no feedback relating to performance or time during the task.

Control procedure: The control procedure consisted of watching a documentary of the same duration as the intervention task. Participants had the choice between all parts of Planet Earth, the complete collection (BBC worldwide, 2006). These documentaries were chosen based on their emotionally neutral yet engaging content. Every 15 min the level of MF was assessed verbally.

### 2.3. Data Collection

#### 2.3.1. Visuomotor Task

**Figure 2:**
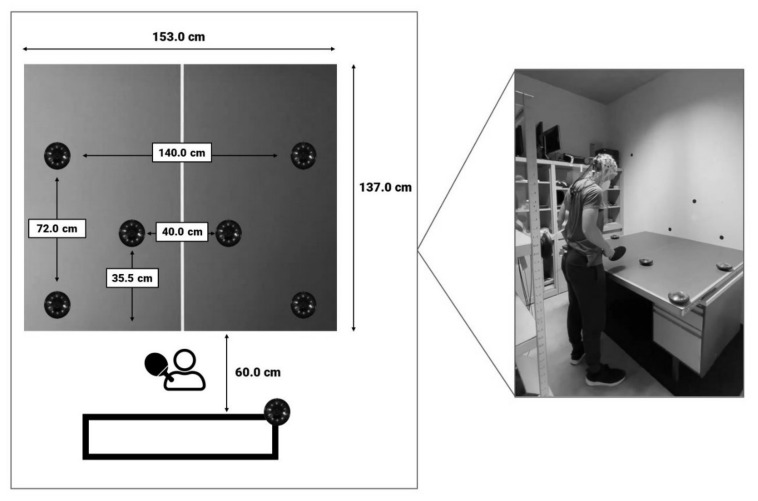
Overview of the setting and design of the visuomotor task.

The visuomotor task was designed in accordance to Van Cutsem et al. (2019) [20]. A specific setting and sequence were designed using Fitlight hardware and software (https://www.fitlighttraining.com/, accessed on 5 October 2020), consisting of eight LED lights. Six of these lights were installed on a table tennis table in a specific manner, while one light was placed behind the participant on the preferred hand site (see Figure 2). The last light was placed in a dark box with a light sensor which was synchronized with the EEG data. The participant was instructed to stand in front of the table and to extinguish one of the six lights by passing a table tennis paddle within 5 cm of the light when it illuminated in either a green, red or yellow color (i.e., simple stimuli). However, when the light turned blue, participants were instructed to ignore the light in front of them and to extinguish the light on their preferred hand site (i.e., complex stimuli). Each color was presented 16 times, for a total of 64 stimuli. The sequence and location in which the colors appeared was programmed randomly (www.randomization.com, accessed on 16 October 2020). The inter-stimulus time of the task varied between 3–6 s, with each time randomly chosen for a total of 16 times. Total task duration was approximately 6 min. Accuracy (i.e., the amount of correct responses) and response time (RT; i.e., the time it took to extinguish the light) for each stimulus type (complex vs. simple stimuli) were calculated based on the data (i.e., a list of accuracy and RT on each stimulus in the visuomotor task) collected by the respective software. Because of the motor simplicity of the present task as opposed to Van Cutsem et al. [20], the accuracy outcome was primarily used to assess inclusion and exclusion of visuomotor RT data, as we expected that missing a certain amount of stimuli can only be linked to an error in stimuli detection. Therefore, if participants missed more than 25% of stimuli, the total data of the participants on the visuomotor task was excluded from further analysis.

#### 2.3.2. EEG Recordings

For EEG recording and data preparation, brain activity was continuously measured throughout the entire duration of the experimental protocol; 32 active gel-based Ag/AgCl electrodes (actiCAP, LiveAMP, Brain Products, Munich, Germany) were attached to the heads of the participants, conforming to the international 10–20 system [36]. Sampling rate was set at 500 Hz and electrode impedance was always kept lower than 10 kΩ throughout the experiment. The beginning of the trial consisted of baseline measurements with 2 min eyes open, and 2 min eyes closed. The program BrainVision Analyzer (Brain Products GmbH, Munich, Germany) was utilised to pre-process the EEG data. The sampling rate was first downsampled to 256 Hz, filtered (high pass: 0.1 Hz (order 8), low pass: 45 Hz (order 8) and Notch: 50 Hz, slope 48 dB/oct) using a Butterworth filter design and re-referenced to an average. EEG data was then divided into specific data sets based on marker positioning (Eyes open, Eyes closed, Fitlight PRE, flanker PRE, flanker POST and Fitlight POST). Artifacts were manually removed using raw data inspection and level triggers were placed based on the specific stimuli of the tasks (i.e., illumination of lights during the Fitlight task, and Flanker task stimuli). An independent component analysis (ICA) and reverse ICA were employed to further reduce periodically recurring artifacts of each specific data set.

For ERP analysis, the N1, P2, N2, and P3b ERPs were assessed across all task data segments (i.e., Stroop, flanker and Fitlight). All information concerning definitions, suspected latencies and electrode locations based on regions of interests (ROI) of the measured ERPs can be found in Table 1. All expected ERPs were visually confirmed. N1 was measured because of its proven susceptibility to MF, association with early visual processing originating from the visual cortex and involvement in spatial attention [31,34,37]. Both P2 and N2 have also been shown to be impacted by MF, and are also believed to originate from the ACC, an area that has been assumed to be the physiological basis of the effect of MF on human performance [25,26,31,32,34]. The P2 is a visual sensory ERP which has been proposed to represent salience detection, the recall of task rules, and allocation of attentional resources [30,31,38]. N2 is an anteriorly located ERP and represents processes of cognitive control, such as conflict monitoring, response inhibition, and error monitoring [32,37]. Lastly, P3b has frequently been connected to (among other cognitive processes) attentional resources, decision making, executive functions and motoric functions, which are all processes that are negatively impacted by MF [18,26,39].

Spectral power analysis: Following the inverse ICA for the continuous EEG data set of interest, segments with a length of 4 s and with an overlap of 2 s were extracted [40]. The resulting data segments were tapered with a Hanning window with 10% of the total segment length. Fast Fourier transform (FFT) power spectra were used to extract power spectral data, with a spectral resolution of 0.25 Hz. These segments were then averaged to stabilize the spectral content. The spectral bands utilized in this study along with their frequency and ROI can be found in Table 1.

#### 2.3.3. Subjective and Physiological Secondary Outcome Measures

Multiple subjective outcome measures were administered to the participants. A pre-test checklist was evaluated at the beginning of each trial to check for any abnormalities which warranted exclusion (e.g., “Did you ingest any beverages containing caffeine this morning?”). The M-VAS was used as a subjective manipulation check. This VAS scale consisted of a 10-cm line which was labelled at one end with ‘not at all mentally fatigued’ and at the other with ‘extremely mentally fatigued’ [41]. Participants were instructed to fill in this questionnaire as they felt at that specific moment. The M-VAS was administered throughout the trial, with nine measurement points (see Figure 1). Additional subjective psychological assessments included the success and intrinsic motivation scales developed by Matthews et al. (2001) [42] and the NASA-TLX [43]. Lastly, RPE was measured using the 15-point (6–20) scale developed by Borg et al. (1982) [44] each time after performing the visuomotor task. Throughout the entire experiment, the heart rate (HR) was measured using a polar SR 400 watch.

### 2.4. Statistical Analysis

All statistical tests were conducted using the Statistical Package for the Social Sciences, version 24 (SPSS Inc., Chicago, IL, USA). All data are presented as means ± standard deviation (SD). Normality was checked using the Shapiro–Wilk test and visually confirmed with histograms. If data were not normally distributed, non-parametrical equivalents were used instead. Mauchly’s test was used to verify for sphericity. However, if sphericity could not be assumed, the Greenhouse–Geisser procedure corrected the significance of F-values. Two-way repeated measures (RM) (2 × 2) effect ANOVAs were conducted to assess the effect of condition (MF vs. CON) and time (PRE vs. POST) on RPE, Matthews intrinsic and success motivation scores, and accuracy and reaction time on the flanker task. A two-way RM (2 × 3) ANOVA was used to assess the effect of condition and time (first to third measurement) on the different subscales of the NASA-TLX. Another two-way RM (2 × 4) ANOVA was used to assess the effect of time (first to fourth block) and stimuli (meaning vs. color) on reaction time and accuracy of the Stroop task. A two-way RM (2 × 9) ANOVA was employed to investigated the effect of condition and time (first to ninth measurement) on the M-VAS and HR measurements. A three-way RM (2 × 2 × 2) effect ANOVA was used to analyse the effect of condition, time and stimuli (simple vs. complex) on the RT of the visuomotor task. A three-way RM ANOVA was adopted to assess the effect of condition, time and ROI on the amplitude and latency of the investigated ERP values during the flanker and Fitlight tasks. By including ROI as a factor, the amount of statistical testing required is significantly reduced. The effect of ROI was not added to the result section in order to reduce irrelevant information, as we were only interested in ROI effects if significant interaction effects were present. The effects of condition, time and ROI on transformed spectral power of theta (θ), lower alfa (α) and upper α bands during the flanker and Fitlight task were investigated using a three-way (2 × 2 × 7) RM ANOVA. If interaction effects between the investigated factors were seen in the three or two-way mixed ANOVAs, subsequent two-way mixed ANOVAs or paired sample t-tests, respectively, were employed to elucidate the main effects of condition and time. If no interaction effect was present, the main effects of condition and time were immediately interpreted using the aforementioned statistical tests and Bonferroni corrections. Partial eta square (η_p_^2^) and Cohen’s d (d) were used as effect sizes. Ranges used for η_p_^2^ were small = 0.01; medium = 0.06; large = 0.14. Ranges used for d were <0.2 = trivial; 0.2–0.6 = small; 0.6–1.2 = moderate; 1.2–2.0 = large; >2.0 = very large. The significance level was set at 0.05.

## 3. Results

### 3.1. Effect of MF on Visuomotor Performance

**Figure 3:**
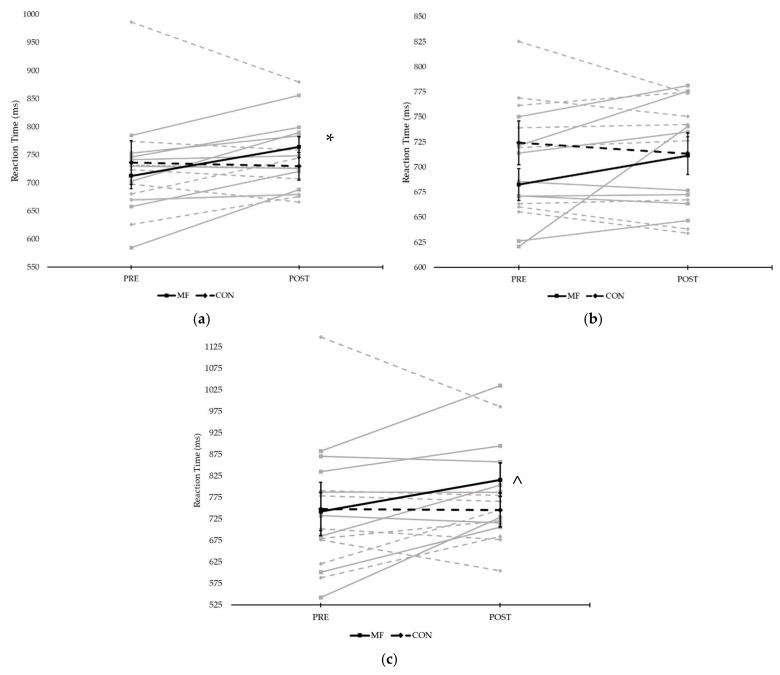
Reaction time on the visuomotor task comparing both conditions pre- and post-experimental/control task in all individuals: (**a**) all stimuli types; (**b**) simple stimuli (red, green and yellow); and (**c**) complex stimuli (blue) (* = significant difference between pre and post in the MF condition; ^ = significant difference between conditions post task; grey lines represent individual responses, bold lines represent means ± SE).

A 2 × 2 × 2 RM ANOVA was conducted to assess the effect of condition, time and stimuli on Fitlight task performance. This analysis showed a significant interaction effect between time and condition (*F*(1,7) = 5.607, *p* = 0.050, η_p_^2^ = 0.445). Subsequent post hoc tests showed a significantly slower visuomotor reaction time from pre- (713 ± 23 ms) to post (764 ± 19 ms) in the MF condition (*F*(1,7) = 14.899, *p =* 0.006, η_p_^2^ = 0.680) for all stimuli; specifically, in the inhibitory stimuli (*t*(7) = 5.199, *p* = 0.001, d = 1.84), a significantly slower visuomotor reaction time post-task in MF (816 ± 111 ms) compared to CON (745 ± 112 ms). Figure 3 displays the effects of condition and time on visuomotor performance.

### 3.2. Visuomotor Task EEG Outcomes

#### 3.2.1. ERP Analysis

There were no effects of time or condition on amplitude or latency of the N2, P3b, P2 and N1 variable during the visuomotor task.

#### 3.2.2. Spectral Band Analysis

Upper α band: There was a significant interaction effect between time and ROI (*F*(1.4,12.5) = 5.825, *p* = 0.024, η_p_^2^ = 0.393). Post-hoc analysis showed a significant interaction between time and condition in the Cz, C3 and C4 electrodes (*F*(1,9) = 15.789, *p* = 0.003, η_p_^2^ = 0.637) and the fusiform gyrus (*F*(1,9) = 6.556, *p =* 0.031, η_p_^2^ = 0.421), while also showing a general effect of time (*F*(1,9) = 6.632, *p =* 0.030, η_p_^2^ = 0.424) in the inferior/orbitofrontal cortex, with the pre-task energy (1.03 ± 0.49 μV^2^) being higher than the post-task energy (0.68 ± 0.12 μV^2^). Paired sample *t*-tests showed a decrease in energy between pre-task (0.65 ± 0.54 μV^2^) and post-task (0.28 ± 0.18 μV^2^) in the MF condition (*t*(9) = 2.712, *p* = 0.024, d = 0.94) in the P7, P8, PO9 and PO10 electrodes only.

Lower α band: an interaction effect between time and ROI was observed (*F*(1.2,10.6) = 1.828, *p* = 0.026, η_p_^2^ = 0.411). Additional analysis of the different ROIs showed an effect of time (*F*(1,9) = 6.348, *p* = 0.033, η_p_^2^ = 0.414), with a higher power pre-test (1.14 ± 0.26 μV^2^) compared to post-test (0.74 ± 0.15 μV^2^) in the F7 electrode, independent of condition.

θ band: a significant interaction effect between time and ROI was found (*F*(1.1,10.1) = 10.735, *p =* 0.007, η_p_^2^ = 0.544). Distinct post-hoc tests examining all ROIs showed a significant effect of time (*F*(1,9) = 11.430, *p* = 0.008, η_p_^2^ = 0.559; PRE = 1.91 ± 0.35 μV^2^; POST = 1.19 ± 0.27 μV^2^) in the F7 electrode, and an effect of condition (*F*(1,9) = 6.592, *p* = 0.030, η_p_^2^ = 0.423; MF = 0.29 ± 0.04 μV^2^; CON = 0.38 ± 0.06 μV^2^) in the Fz, F3 and F4 electrodes.

### 3.3. Secondary Outcome Measures

#### 3.3.1. NASA-TLX

Table 2 shows the effects of both condition and time on the different subscales of the NASA-TLX. Regarding condition, there was only a significant difference in frustration in the second time point (*p* = 0.007), with MF causing a higher frustration compared to CON. The effects of time are also presented in the table, with an additional effect of time on frustration in the MF condition.

#### 3.3.2. Motivation

A significant interaction effect (*F*(1,10) = 12.692, *p* = 0.005, η_p_^2^ = 0.559) between condition and time in the intrinsic motivation scale was observed. Post hoc paired sample *t*-tests showed a significant difference between pre (22.09 ± 2.17) to post (19.27 ± 2.94) in the MF condition (*t*(10) = 3.492, *p* = 0.006, d = 1.05) and between MF and CON (19.55 ± 3.08) in the pre-task time interval (*t*(10) = 3.088, *p* = 0.011, d = 0.93). There were no significant effects for either time or condition on the success motivation scale.

#### 3.3.3. Rate of Perceived Exertion

A 2 × 2 RM ANOVA showed a significant effect of time (*F*(1,10) = 8.711, *p* = 0.014, η_p_^2^ = 0.466) and condition (*F*(1,10) = 11.011, *p* = 0.008, η_p_^2^ = 0.524) in RPE measures, without a significant interaction effect. Post hoc Bonferonni tests showed a significant increase from pre (8.36 ± 0.50) to post (M ± SD = 9.55 ± 0.58; *p* = 0.014) experimental/control task, and a significantly higher value for RPE in the control condition (9.59 ± 0.56) compared to the MF condition (8.32 ± 0.51).

#### 3.3.4. Heart Rate

Analysis of HR values showed no significant effect of condition. However, there was a significant effect of time (*F*(8,56) = 5.106, *p* < 0.001, η_p_^2^ = 0.422), with post hoc analysis showing that these significant effects can be contributed to the significant drop in HR in measures 4, 5, 6, 7 (during and right after the Stroop task) and 8 (right after the post-flanker task) compared to the first time point (at the beginning of the experiment).

### 3.4. Manipulation Checks

#### 3.4.1. Subjective (M-VAS)

**Figure 4:**
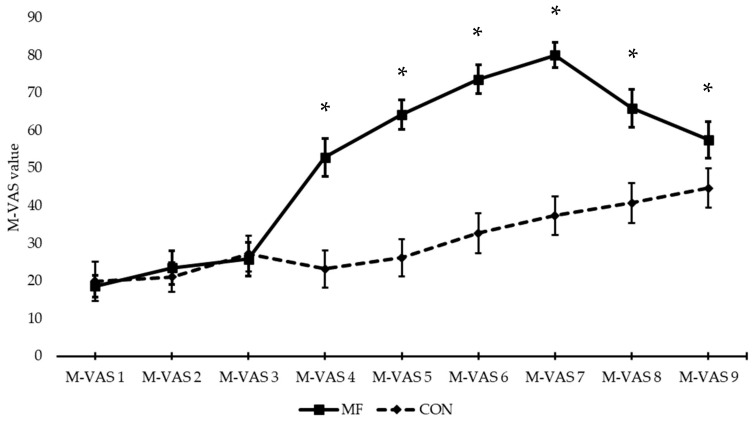
Graph of the M-VAS measurements across conditions (graph lines represent means ± SE;* = significant difference between the conditions).

A significant interaction effect between condition and time (*F*(3,30) = 263.402, *p* < 0.001, η_p_^2^ = 0.701) was present. This interaction indicated that, in terms of the effect of time, the subjective experience of MF increased in both the MF (*F*(3,26) = 42.970, *p* < 0.001, η_p_^2^ = 0.811) and the CON (*F*(3,33) = 13.497, *p <* 0.001, η_p_^2^ = 0.574) condition. MF significantly increased during the Stroop task (from M-VAS 4 onward (first M-VAS administered during the Stroop task)), and also significantly decreased afterwards (comparison between M-VAS 7 (M-VAS administered immediately after the Stroop task) and 8 (M-VAS administered immediately after the post-flanker task)) (Table 3). In the control condition, the increased MF occurs more slowly (only significant in comparison between M-VAS 1 and 8/9) and there is no significant decrease in MF sensation afterwards. Paired sample t-tests at every time point showed that there was a significant difference in MF from M-VAS 4 onward between the MF and CON condition (Figure 4/Table 4).

Analysis of reaction time on the different blocks of the Stroop task showed no effect of time (*F*(1,13) = 0.422, *p* = 0.587, η_p_^2^ = 0.040) or stimuli (*F*(1,10) = 2.562, *p* = 0.141, η_p_^2^ = 0.204). For accuracy variables, two Friedmann tests showed no effect of time regardless of type of stimuli. Four Wilcoxon signed rank tests (one for every block) showed significant differences in all four blocks between the type of stimuli, with a significantly higher accuracy when performing the simple stimuli compared to the complex stimuli.

**Figure 5:**
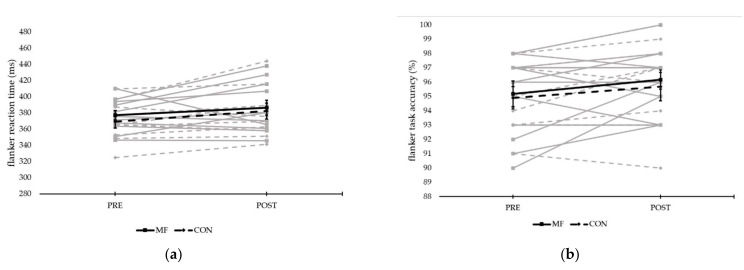
Reaction time and accuracy of the flanker task comparing both conditions pre- and post-experimental/control task in all individuals: (**a**) reaction time (ms); and (**b**) accuracy (%) (grey lines represent individual responses, bold lines represent means ± SE).

There was no effect of condition or time on flanker reaction time (see Figure 5). There was also no effect of condition on flanker accuracy. There was, however, an effect of time on the accuracy of the flanker task (*F*(1,9) = 5.299, *p* = 0.047, η_p_^2^ = 0.371), with the accuracy post-task (96 ± 1%) being higher than pre-task (95 ± 1%).

#### 3.4.2. Physiological (EEG)

SPA: There was no effect of time or condition on upper and lower α and θ frequency band during the flanker task.

ERP: There was a significant effect of condition on the amplitude of the N1 ERP (*F*(1,7) = 6.724, *p* = 0.036, η_p_^2^ = 0.490), with the amplitude of the MF condition (−1.05 ± 0.21 µV) being lower than the amplitude of the control condition (−1.29 ± 0.26 µV). No effects were found on the latency of the N1 variable. The opposite was true for the P2 ERP, showing an effect of time on latency (*F*(1,7) = 8.018, *p* = 0.025, η_p_^2^ = 0.534), but no effects on amplitude. This effect of time consisted of a shorter latency pre-task (157 ± 4 ms) compared to post-task (161 ± 4 ms). Due to a significant three-way interaction effect between time, condition and ROI on amplitude of P3b (*F*(1.1,7.7) = 9.306, *p* = 0.015, η_p_^2^ = 0.571), subsequent post hoc tests were carried out. These tests revealed a significant interaction effect between time and ROI within the MF condition (*F*(2,14) = 13.061, *p* = 0.001, η_p_^2^ = 0.651) and a significant interaction effect between condition and ROI pre-task (*F*(1.1,7.8) = 6.318, *p* = 0.034, η_p_^2^ = 0.474). Paired sample t-tests pointed to decreases in amplitude between pre-task (3.75 ± 1.99 µV) and post-task (1.40 ± 2.13 µV) in the MF condition (*t*(7) = 3.607, *p* = 0.009, d = 1.14) and between conditions (CON = 1.73 ± 1.52 µV) pre-task (*t*(7) = 2.743, *p* = 0.029, d = 1.14), with both effects only present in the fusiform gyrus. There were no effects of time or condition on P3b latency. There were no effects of time or condition on amplitude and latency of N2.

## 4. Discussion

### 4.1. Summary of the Findings

The results of the present study show that MF negatively affects the reaction time of visuomotor performance, especially inhibitory responses. Meanwhile, the visuomotor task showed differences in EEG outcomes due to MF exclusively related to frequency bands, and no effects of MF on ERPs during the visuomotor task were observed. The differences in frequency bands amount to overall decreases in brain activity in the MF condition. Secondary outcome measures remained mostly uninfluenced.

### 4.2. Effect of Mental Fatigue on Visuomotor Performance in Trained Table Tennis Players

An increase in reaction time was observed from pre-Stroop to post-Stroop in the MF condition. Moreover, there was a significant difference in reaction time between both conditions (post-Stroop/documentary) when examining the inhibitory stimuli, with subjects who experienced MF having a higher reaction time compared to the control condition. These results are in line with the previous study of Van Cutsem et al. [20], on which the visuomotor task of the present study is primarily based. Specifically, an increase in reaction time of 7% was found when comparing the data of the inhibitory stimuli pre- and post-Stroop task in the MF condition [20]; in comparison, the present study noted an increase of 9%. As the authors [20] mentioned, this is to be expected, as the effects of MF on cognitive performance are attributed to a decrease in executive control and attention, which makes it more difficult to deviate from automatic responses and focus on the task at hand [45,46]. This study, therefore, further adds to the theories of Baumeister et al. [47], who suggested that prolonged mental demand exhausts self-control resources, which impairs executive functions, inhibiting specific processes and functional connectivity in the brain [48]. More specifically, MF might decrease allocation of neural resources to specific areas responsible for stimuli processing, fabricating slower reaction times [27]. Therefore, the effect of MF on the reaction time of inhibitory stimuli is consistent with the literature and confirms accepted knowledge of theoretical MF mechanisms.

Research has suggested that elite athletes might have superior inhibitory control, which could provide them with a higher resistance to MF and its detrimental effects on performance [49]. Moreover, table tennis players exhibit greater cognitive skills such as executive control, which can theoretically be linked to MF-resistance [9,13]. The lack of effect on the behavioral manipulation checks in the present study appear to confirm this statement. Nonetheless, the present study still showed a decrement in visuomotor performance because of MF, despite the training level of the included subjects. Recent research suggests that the supposed resistance to MF that trained athletes exhibit is vague at best, and different subsequent studies imply that this might differ between athletes [20]. A suggestion for this is that athletes are perhaps more resistant, but not immune to MF [20]. Indeed, research has already suggested that there are interindividual differences in the response to MF [50], and that specific subgroups can be constructed based on susceptibility of MF [51]. It is, however, grossly simplistic to imply that these differences are solely the result of training level; other factors such as task representativeness [18], age [52], and genetics [53] could influence the effects of MF on human performance. The confidence intervals of the present study also show that there were widely differing responses to MF, which underlines the importance of interindividual responses to MF. To summarize, the observed effects of MF on visuomotor task reaction time indicate that trained athletes can be affected by MF, and that other factors influence the interindividual responses to MF. These factors certainly remain interesting to consider for further investigation. However, it should still be noted that MF impaired the general reaction time and the reaction time on the inhibitory stimuli post-task, and therefore impaired performance.

### 4.3. Underlying Mechanisms of the Effect of Mental Fatigue on Visuomotor Performance

#### 4.3.1. Neurophysiological

The present study investigated two major EEG outcomes, SPA (i.e., transformation of EEG data into spectral power [28]) and ERPs (i.e., differences in amplitude and latencies potential related to certain events [37]). A recent review by Tran et al. [28] investigating MF using SPA showed that MF generally increases brain activity. More specifically, increases are observed in theta activity in frontal, central and posterior sites and alpha activity in central and posterior sites [28]. These frequencies can be linked to inhibition control and decreased alertness, which indicates that MF makes it more difficult to maintain focus on the task at hand, which contributes to less efficient performance [28,29,54]. Tran et al. [28] concluded that an increase in theta wave activity can be seen as a valid and definite neurophysiological marker for MF. However, the SPA in the present study provided us with unexpected results: a decrease in power over time for the F7 electrode across all bands (PRE > POST in upper and lower α, and PRE < POST in θ), an effect of time in the upper α band across all ROIs (PRE > POST) in the MF condition, and an effect of condition in the Fz, F3 and F4 electrodes (MF < CON) for θ activity. Overall, a decrease in power was found both in the MF condition and generally over time. These reductions in power are consistent with EEG in motion literature, where these decreases reflect an overload of incoming stimuli and a general state of cortical desynchronization during complex movements [55]. This further reduction between tasks might indicate that MF increases this desynchronization between brain regions. Moreover, recent research points out that the increases in power due to MF are often found in task-unrelated intervals, and that there are decreases in specific power bands during task-related activity [56]. The review of Tran et al. [28] only mentions effects of MF within the same task, and does not provide any results of the trade-off between tasks. Lastly, since boredom might play a role in the results, this could have affected the overall power of the specific bands, especially the ones showing a general effect of time, since boredom decreases frequencies in SPA [57]. Therefore, the results could also suggest that MF caused less task engagement when performing the visuomotor task, leading to a decrease in performance. Moreover, within θ power, the Fz, F3 and F4 electrodes were implicated in a decrease in energy. These electrodes are located near the dorsolateral prefrontal cortex, further adding to the literature implicating this region in MF and its effects [25,58].

A drawback of the SPA technique is that the analysis provides only an average change in frequency over a given time period, which fails to utilise the most important benefit of EEG measurements, namely an excellent temporal resolution [59]. Studies investigating the effect of MF on ERP values are scarce, but are possibly more important in the search for explanations behind MF mechanisms as ERPs represent an immediate change in brain activity due to a task stimulus [37]. These types of analyses make use of the temporal properties of EEG, meaning that a change in latency and amplitude of ERPs due to MF can be connected to behavioural indices of MF, which might provide an explanation for these changes in behaviour. There are different types of ERPs based on different cognitive processes, such as N2 (conflict monitoring) and P3b (attentional resources), and preliminary research suggests a decrease in amplitude due to MF, meaning less activation of these specific processes [26,27]. The influence of MF on ERPs could, however, go either way in the presence of performance measures [26,33]. Lower amplitude means less activation of cognitive processes and can be linked to decrements in performance [26]. Meanwhile, a higher amplitude might also be possible, certainly if performance remains unaltered, as this might indicate an increase in cognitive resource division to maintain performance when vigilance decreases [33]. Unfortunately, no significant differences were found regarding the ERPs during the visuomotor task. This could be attributed to the nature of the utilised task. The primary difference between the mentioned studies showing a decrease in amplitude due to MF and the present study is that these declines were observed during the prolonged cognitive task. The high-intensity nature of the visuomotor task resulted in a remarkable increase in the difficulty of identifying ERP values, which resulted in less data to clearly identify. This is further confirmed by the fact that there were significant differences in ERP values in the flanker task. The decreases in amplitude (N1 and P3b) there could be contributed to a decrease in vigilance due to the nature of the employed tasks [33]. Another explanation regarding the non-effects of ERPs can possibly be found in the inability to divide the ERPs based on stimulus type, since we failed to distinguish between the different types of stimuli using the light sensor. Since the inhibitory stimuli were primarily impacted in the visuomotor task, it could be argued that an effect could have been observed in these specific ERPs.

#### 4.3.2. Subjective

Several authors also connect the effects of MF on performance to a decrease in motivation. MF causes decreased motivation, which in turn leads to less attention, and responses that are not automatic are therefore impaired [60,61]. Studies show that if motivation is altered under a mentally fatigued state, performance can be better maintained [60]. This might also be indicated in the present study, as in the MF condition subjects experienced a decrease in intrinsic motivation from pre to post. However, recent research suggests that motivation might not be as closely related to MF as previously thought [62]. Brain related measures point out that subjective MF is not associated with the motivational network of the brain, but rather with a decrease in activation of task-specific circuits [62]. Motivation is also subject to variability, as shown by the significant difference between conditions at the start of the experiments. The reason for performance decrements might therefore lie elsewhere.

A last measure frequently associated with MF is RPE, since an increase in RPE due to MF was seen in the original review by Van Cutsem et al. [17]. This increase in RPE is linked to performance deteriorations under MF: participants perceive the task as more effortful, and will therefore more easily forfeit the task [17]. Most studies examining sport-specific performance and MF effects also asses RPE after physical tasks [18]. The present study also investigated RPE, and found an increase over time, independent of condition, and an opposite than expected effect of condition, where the control group indicated a higher RPE value than the MF group. This effect of time can be explained by the fact that the perception of MF also increased in the control group and that participants in that group already perceived the Fitlight task as more effortful before the experimental/control condition (which could be attributed to day-to-day variability). The review of Habay et al. [18] already showed that the influence of RPE within the effect of MF on sport-specific psychomotor performance remains ambiguous. Moreover, recent research on RPE within MF and open skilled sports suggests that there might not be any effect of MF on RPE in individual sport sessions [63]. Therefore, these results seem to indicate that RPE might play a smaller role in acute MF effects than previously assumed.

### 4.4. Results and Importance of Manipulation Checks

An interesting observation of this study was the increase in subjective feelings of MF in the control task over time. Mangin et al. [64] recently emphasized the importance of a good control task, showing that unsuited tasks increase the MF/ego depletion effect, which in turn might cause an insignificant effect of condition in different studies. The results of the present study show that the documentary increased the subjective MF experience, while there was no detectable effect of condition on the behavioural manipulation checks, and there were no significant differences in mental workload between the different conditions after the experimental/control task. As O’Keeffe et al. [65] mentioned, arousal and boredom are two very important aspects that must be considered when choosing a mentally fatiguing/control task. Both impact performance in healthy individuals [65], therefore the intervention task should not be too boring, and the control task should be emotionally neutral. When Smith et al. [41] compared three different tasks and their effect on indicators of MF, they found that increasing challenge, as opposed to monotony, might result in higher task engagement and thus higher levels of MF. With this in mind, the individualised Stroop task of the present study was designed to challenge individuals and keep arousal high. However, the observed increase in frustration suggests that more alterations to the task may be required. The findings mentioned above emphasize that the choice of both experimental and control task is of utmost importance when designing a MF study. Nevertheless, even if the employed tasks have some flaws, we still conclude that our subjects were more mentally fatigued in the MF condition, based on the M-VAS increases over time in this condition and the significant differences between conditions from the onset of the intervention. The fact that behavioural checks were unaffected in this study might be linked to the training level and/or inter-individual variability in subjects [41]. Moreover, research shows that the M-VAS remains the most sensitive way to assess MF [41].

Definitive evidence of the mentally fatigued state of participants can be found in the physiological manipulation checks, showing a decrease in N1 and P3b amplitude (for P3b, only in the P7 electrode) due to condition effects. These results are consistent with the literature [34]. As Jacquet et al. [34] point out, the overall decrease in N1 amplitude reflects an increase in mental workload and can be used as an indicator of MF. Moreover, the fact that the P3b amplitude only decreased in the fusiform gyrus might also give an indication of the reason behind the behavioural performance maintenance under MF. P3b is often implicated in attention circuits, and a decrease in amplitude is often connected to MF performance decrements [33,34,66]. The fact that this decrease in amplitude only occurs in one ROI (as opposed to the other 6) might indicate that cognitive resources were utilised to maintain task performance in the other brain regions while countering the effects of MF on behavioural performance in the flanker task [34].

As a last confirmation of the mentally fatigued state, the performance of the different subjects was still negatively impacted by MF, as seen by the effects on the visuomotor task. Therefore, it can be concluded that MF was successfully induced. This study further emphasizes the necessary implementation of the three different types of manipulation checks (i.e., subjective, behavioural and (neuro)physiological) in future MF studies [18].

### 4.5. Limitations and Future Directions

Some limitations concerning the present study should be mentioned. Some data were lost in the visuomotor task. This loss in data could have impacted the results, and should be taken into consideration when interpreting the present data. However, the confirmation that MF affects visuomotor performance, also determined by other studies [20], seems to indicate that this influence was minimal. The discussion surrounding the tasks employed in the present study and the decreases in spectral power mention that boredom and arousal play an essential role in MF. Measures of boredom (such as a Boredom-VAS) and arousal (such as the Brunel Mood scale) were not employed, and therefore no definitive conclusion surrounding these facts can be made. Lastly, the ability to divide the ERPs based on stimulus type might have allowed us to further comment on the mechanisms surrounding the declines in visuomotor performance due to MF. However, despite these limitations, the present study provides further confirmation that MF negatively affects sport-specific visuomotor performance.

As mentioned before [18], more studies, especially testing fundamental theories, should attempt to include EEG in their experimental protocol when assessing effects of MF in order to further elucidate the mechanisms of this complex phenomenon.

The present study provides further proof that the current visuomotor task is a valid way to assess visuomotor performance decrements due to MF. As Van Cutsem et al. [20] mentioned, this kind of task might provide coaches with a more practical tool (as opposed to purely cognitive tasks) to evaluated possible MF-related resistance in their athletes.

### 4.6. Practical Implications

The present study provides new proof that MF affects sport-specific psychomotor performance in healthy athletes, as shown in the review of Habay et al. [18]. Specifically for TT athletes, we now know that MF impairs not only technical [16] but also sport-specific visuomotor performance. Since TT is a sport with high mental and physical demands, different countermeasures such as caffeine [30] and creatine [67] could be used to combat acute MF during practice and competitions. Moreover, research also points out that training might make individuals more resistant to MF [68]. Other ways to combat MF might have more to do with competition organisation, such as travel [69]. Coaches, trainers and players employed and participating in TT need to be aware of the definition and effects of and countermeasures against MF in order to better protect the performance of their athletes. Future studies should try to examine the benefits of specific training or countermeasures to combat MF in open skill sports [70].

## 5. Conclusions

The present study provides clear evidence that the visuomotor performance of trained TT players is negatively impacted by MF, further adding to knowledge regarding the effects of MF on visuomotor performance [18,20]. Specifically, MF worsened reaction time in the inhibitory stimuli, while the spectral analysis pointed to an increased desynchronization of brain regions. These findings further prove the impact of MF on TT-specific performance [16], suggesting that coaches and other personnel employed in this sport should be aware of MF, its effects, and possible ways to counteract it. Future research should build on these findings while including EEG measurements in analysis of the data and providing clear proof of the mentally fatigued state of participants by using multiple valid manipulation checks.

## Figures and Tables

**Table 1:** Definitions, localisations and suspected latencies of Spectral power and ERPs of interest.

Event Related Potentials									
Variable	Definition	Suspected Latency	Regions of Interest						
			DLPC	PC	PMC	IOC	AG	FG	SAC
N1	First negative going peak	90–150 ms	X	X	X				
P2	Second positive going peak	80–260 ms	X			X			
N2	Second negative going peak	200–315 ms	X			X			
P3b	Third and largest positive going peak	280–450 ms					X	X	X
**Spectral Bands**									
**Symbol**	**Name**	**Frequency**	**Regions of Interest**						
			**DLPC**	**PC**	**PMC**	**IOC**	**AG**	**FG**	**SAC**
θ	Theta	4–<8 Hz	X	X	X	X	X	X	X
Lα	Lower alpha	8–<10 Hz	X	X	X	X	X	X	X
Uα	Upper alpha	10–<13 Hz	X	X	X	X	X	X	X

DLPC = dorsolateral prefrontal cortex (Fz, F3 and F4), PC = premotor cortex (FC1 and FC2), PMC = primary motor cortex (Cz, C3 and C4), IOC = inferior/orbitofrontal cortex (F7), AG = angular gyrus (P3 and P4), FG = fusiform gyrus (P7, P8, PO9 and PO10), SAC = somatosensory association cortex (Pz).

**Table 2:** Effects of condition and time on the different subscales of the NASA-TLX.

Scale	Mean ± SD				Effect of Condition	Effect of Time	Post Hoc Time		
		Time 1	Time 2	Time 3			1 ↔ 2	2 ↔ 3	1 ↔ 3
Mental Workload	MF	40 ± 24	69 ± 28	52 ± 28	*p* = 0.120	*p* = 0.004 *	*p* = 0.028 *	*p* = 0.039 *	*p* = 0.549
	CON	39 ± 28	53 ± 26	44 ± 22	η_p_^2^ = 0.224	η_p_^2^ = 0.418			
Physical Workload	MF	11 ± 10	7 ± 5	14 ± 7	*p* = 0.093	*p* = 0.002 *	*p* = 0.096	*p* = 0.004 *	*p* = 0.420
	CON	15 ± 12	7 ± 6	21 ± 17	η_p_^2^ = 0.256	η_p_^2^ = 0.469			
Tempo	MF	34 ± 25	56 ± 21	43 ± 24	*p =* 0.054	*p* = 0.005 *	*p* = 0.023 *	*p* = 0.170	*p* = 0.360
	CON	31 ± 22	49 ± 31	35 ± 25	η_p_^2^ = 0.323	η_p_^2^ = 0.410			
Performance	MF	41 ± 16	44 ± 14	41 ± 12	*p* = 0.057	*p* = 0.904	/	/	/
	CON	35 ± 9	36 ± 16	37 ± 12	η_p_^2^ = 0.316	η_p_^2^ = 0.010			
Effort	MF	36 ± 14	58 ± 22	42 ± 22	*p* = 0.394	*p* = 0.007 *	*p* = 0.023 *	*p* = 0.076	*p* = 1.000
	CON	37 ± 22	47 ± 25	40 ± 13	η_p_^2^ = 0.074	η_p_^2^ = 0.388			
Frustration	MF	11 ± 9	51 ± 23	36 ± 21	1: *p* = 0.160 d = −0.457 2: *p* = 0.007 * d = 1.008 3: *p* = 0.423 d = 0.847	MF: *p* < 0.001 * η_p_^2^ = 0.562 CON: *p* = 0.276 η_p_^2^ = 0.121	*p* = 0.002 *	*p* = 0.196	*p* = 0.039 *
	CON	17 ± 10	23 ± 23	27 ± 24			/	/	/

* = significant difference (*p* < 0.05).

**Table 3:** Effect of time on the M-VAS measurements in both conditions.

Mental Fatigue				Control			
M-VAS	Mean Diff.	95% CI	*p*	M-VAS	Mean Diff.	95% CI	*p*
1 ↔ 2	−5.0	[−18.4; 8.6]	1.000	1 ↔ 2	−1.2	[−17.0; 14.6]	1.000
1 ↔ 3	−7.2	[−25.0; 10.6]	1.000	1 ↔ 3	−7.4	[−30.1; 15.4]	1.000
1 ↔ 4	−34.3	[−57.0; −11.6]	0.002 *	1 ↔ 4	−3.3	[−24.8; 18.3]	1.000
1 ↔ 5	−45.6	[−67.8; −23.5]	<0.001 *	1 ↔ 5	−6.3	[−28.4; 15.8]	1.000
1 ↔ 6	−55.0	[−78.4; −31.6]	<0.001 *	1 ↔ 6	−12.8	[−30.3; 4.7]	0.340
1 ↔ 7	−61.5	[−83.7; −39.2]	<0.001 *	1 ↔ 7	−17.5	[−36.7; 1.8]	0.094
1 ↔ 8	−47.4	[−73.8; −20.9]	0.001 *	1 ↔ 8	−20.8	[−40.9; −0.8]	0.038 *
1 ↔ 9	−38.9	[−66.8; −11.1]	0.004 *	1 ↔ 9	−24.8	[−49.5; −0.1]	0.049 *

* = significant difference between time point 1 and the present time point.

**Table 4:** M-Vas values across time point in both conditions with statistical comparison.

M-Vas	1	2	3	4	5	6	7	8	9
MF	18 ± 10	24 ± 15	26 ± 15	53 ± 17	64 ± 13	73 ± 13	80 ± 11	66 ± 17	58 ± 16
CON	20 ± 17	21 ± 13	27 ± 16	23 ± 16	26 ± 17	33 ± 18	37 ± 17	41 ± 18	45 ± 18
*p*	0.807	0.612	0.756	0.001 *	<0.001 *	<0.001 *	<0.001 *	0.003 *	0.021 *

* = significant difference between the conditions; M-VAS 1= baseline; M-VAS 2= right after the first Fitlight task; M-VAS 3 = right after the first flanker task, just before the start of the Stroop task; M-VAS-4,5,6 = throughout the Stroop task; M-VAS 7 = at the end of the Stroop task; M-VAS 8 = right after the second flanker task; M-VAS 9 = at the end of the second Fitlight task.3.4.2. Behavioural (Stroop and Flanker).

## Data Availability

The data presented in this study are available on request from the corresponding author.
