# Correction: Habay et al. Mental Fatigue-Associated Decrease in Table Tennis Performance: Is There an Electrophysiological Signature? *Int. J. Environ. Res. Public Health* 2021, *18*, 12906

**DOI:** 10.3390/ijerph20032545

**Published:** 2023-01-31

**Authors:** Jelle Habay, Matthias Proost, Jonas De Wachter, Jesús Díaz-García, Kevin De Pauw, Romain Meeusen, Jeroen Van Cutsem, Bart Roelands

**Affiliations:** 1 Human Physiology and Sports Physiotherapy Research Group, Faculty of Physical Education and Physiotherapy, Vrije Universiteit Brussel, Pleinlaan 2, 1050 Brussels, Belgium; 2 BruBotics, Vrije Universiteit Brussel, 1050 Brussels, Belgium; 3 Faculty of Sport Sciences, University of Extremadura, 10003 Caceres, Spain; 4 Vital Signs and Performance Monitoring Research Unit, LIFE Department, Royal Military Academy, Avenue de la Renaissancelaan 30, 1000 Brussels, Belgium

## Additional Affiliation

In the published publication [1], there was an error regarding the affiliation(s) for Jelle Habay, Matthias Proost, Jonas DeWachter, Romain Meeusen and Bart Roelands. In addition to affiliation 1, it should be 1 and 2. Affiliation 2 and 3 exchange order.

## Error in Table

In the original publication, there was a mistake in Table 1. Definitions, localisations and suspected latencies of spectral power and ERPs of interest as published. In the table, it is noted that upper alpha can be found within the frequencies 4–<8 Hz and that theta can be found in 10–<13 Hz. However, this in incorrect and should be the other way around. The correct classification should be that the spectral band theta is between the frequencies 4 and <8 Hz and the spectral band upper alpha is between 10 and <13 Hz. The corrected Table 1. Definitions, localisations and suspected latencies of spectral power and ERPs of interest appears below. The authors apologize for any inconvenience caused and state that the scientific conclusions are unaffected. This correction was approved by the Academic Editor. The original publication has also been updated.

## Figures and Tables

**Table 1:** Definitions, localisations and suspected latencies of Spectral power and ERPs of interest.

Event Related Potentials									
Variable	Definition	Suspected Latency	Regions of Interest						
			DLPC	PC	PMC	IOC	AG	FG	SAC
N1	First negative going peak	90–150 ms	X	X	X				
P2	Second positive going peak	80–260 ms	X			X			
N2	Second negative going peak	200–315 ms	X			X			
P3b	Third and largest positive going peak	280–450 ms					X	X	X
**Spectral Bands**									
**Symbol**	**Name**	**Frequency**	**Regions of Interest**						
			**DLPC**	**PC**	**PMC**	**IOC**	**AG**	**FG**	**SAC**
θ	Theta	4–<8 Hz	X	X	X	X	X	X	X
Lα	Lower alpha	8–<10 Hz	X	X	X	X	X	X	X
Uα	Upper alpha	10–<13 Hz	X	X	X	X	X	X	X

DLPC = dorsolateral prefrontal cortex (Fz, F3 and F4), PC = premotor cortex (FC1 and FC2), PMC = primary motor cortex (Cz, C3 and C4), IOC = inferior/orbitofrontal cortex (F7), AG = angular gyrus (P3 and P4), FG = fusiform gyrus (P7, P8, PO9 and PO10), SAC = somatosensory association cortex (Pz).

## References

[1] Habay J., Proost M., De Wachter J., Díaz-García J., De Pauw K., Meeusen R., Van Cutsem J., Roelands B. (2021). Mental Fatigue-Associated Decrease in Table Tennis Performance: Is There an Electrophysiological Signature?. Int. J. Environ. Res. Public Health.

